# Mechanisms of Action of Adjuvants

**DOI:** 10.3389/fimmu.2013.00114

**Published:** 2013-05-16

**Authors:** Sunita Awate, Lorne A. Babiuk, George Mutwiri

**Affiliations:** ^1^Vaccine and Infectious Disease Organization-International Vaccine Centre, School of Public Health, University of SaskatchewanSaskatoon, SK, Canada; ^2^Vaccinology and Immunotherapeutics program, School of Public Health, University of SaskatchewanSaskatoon, SK, Canada; ^3^University of AlbertaEdmonton, AB, Canada

**Keywords:** adjuvants, mechanisms, innate immunity, cell recruitment and activation, inflammasomes, antigen presentation, dendritic cells

## Abstract

Adjuvants are used in many vaccines, but their mechanisms of action are not fully understood. Studies from the past decade on adjuvant mechanisms are slowly revealing the secrets of adjuvant activity. In this review, we have summarized the recent progress in our understanding of the mechanisms of action of adjuvants. Adjuvants may act by a combination of various mechanisms including formation of depot, induction of cytokines and chemokines, recruitment of immune cells, enhancement of antigen uptake and presentation, and promoting antigen transport to draining lymph nodes. It appears that adjuvants activate innate immune responses to create a local immuno-competent environment at the injection site. Depending on the type of innate responses activated, adjuvants can alter the quality and quantity of adaptive immune responses. Understanding the mechanisms of action of adjuvants will provide critical information on how innate immunity influences the development of adaptive immunity, help in rational design of vaccines against various diseases, and can inform on adjuvant safety.

## Introduction

The goal of vaccination is induction of protective immunity and in some vaccines this can be enhanced by addition of adjuvants. Adjuvants (Latin word *adjuvare*, meaning “to help or aid”) were first described by Ramon as “substances used in combination with a specific antigen that produced a more robust immune response than the antigen alone” (Ramon, 1924). Many diverse classes of compounds have been assessed as adjuvants including mineral salts, microbials products, emulsions, saponins, cytokines, polymers, microparticles, and liposomes (Guy, 2007). Based on their proposed mechanisms of action, vaccine adjuvants have been broadly divided into delivery systems and immuno-stimulatory adjuvants (Singh and O’Hagan, 2003). In general, delivery systems were previously thought to act by providing a depot while immuno-stimulatory adjuvants activate cells of the innate immune system (Pashine et al., 2005). However, this classification is no longer appropriate since now there is evidence that some delivery systems can activate innate immunity.

**Figure 1:**
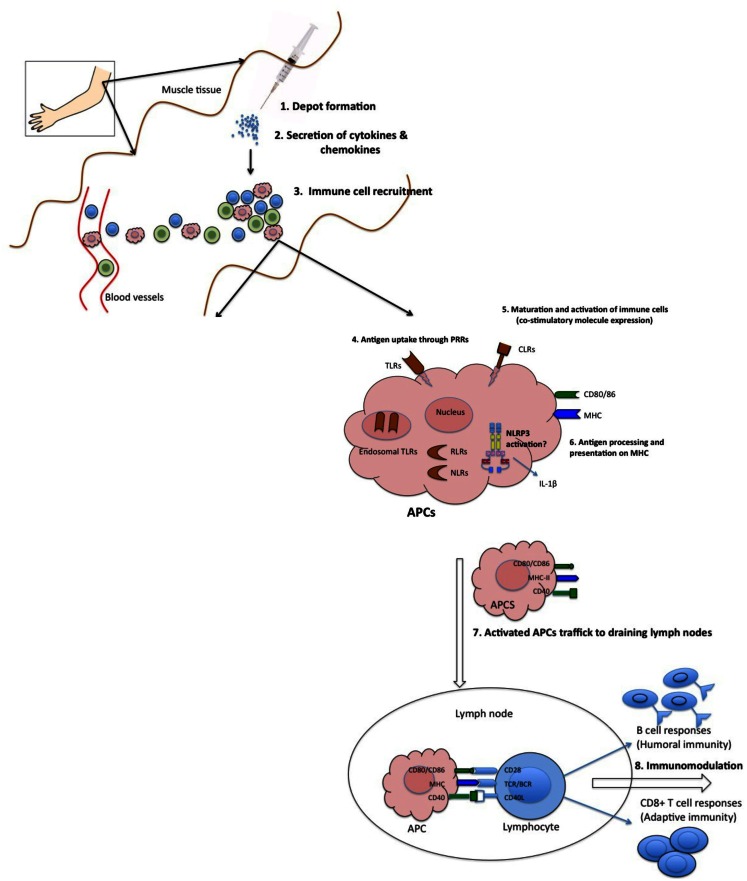
**Proposed mechanisms of action of adjuvants**. (1) Some adjuvants presumably form a depot at the site of injection, which is associated with slow release of antigen. (2) Other adjuvants are associated with transient secretion of cytokines and chemokines. (3) Secreted cytokines and chemokines are involved in recruitment of various immune cells to the injection site. These recruited cells secrete cytokines and chemokines, in turn attract other immune cells. All these events lead to formation of a local immuno-competent environment at the injection site. (4) The recruited APCs express various PRRs both on the surface (TLRs, CLRs) and intracellularly (NLRs and RLRs), which are recognized and/or are activated by the adjuvants. (5) This leads to maturation and activation of recruited APCs. Mature APCs up-regulate the expression of MHC and co-stimulatory molecules. (6) They are also characterized by increased capacity for antigen processing and presentation. (7) Mature APCs then migrate to the draining lymph nodes to interact with antigen-specific B or T cell to (8) activate potent antibody secreting B cells and/or effector CD8^+^ T cell responses.

Surprisingly, despite the wide use of vaccine adjuvants in billions of doses of human and animal vaccines, the mechanisms of action by which they potentiate immune responses are not well characterized. This is well captured in a famous quote by Janeway (1989) who observed that adjuvants are “the immunologists’ dirty little secret.” However, recent advances in immunobiological research have revealed several mechanisms by which adjuvants act. Available evidence suggests that adjuvants employ one or more of the following mechanisms to elicit immune responses: (1) sustained release of antigen at the site of injection (depot effect), (2) up-regulation of cytokines and chemokines, (3) cellular recruitment at the site of injection, (4) increase antigen uptake and presentation to antigen presenting cells (APC), (5) activation and maturation of APC [increased major histocompatibility complex (MHC) class II and co-stimulatory molecules expression] and migration to the draining lymph nodes, and (6) activation of inflammasomes (Figure 1) (Cox and Coulter, 1997; Hoebe et al., 2004; Fraser et al., 2007). In this review, we will address the proposed mechanisms of action of vaccine adjuvants with specific emphasis on licensed adjuvants (Table 1).

**Table 1:** **Mechanisms of action of adjuvants licensed for human use**.

Adjuvants	Proposed mechanisms of action	Immune response activated	Licensed vaccines	Reference
Alum	No depot effect	↑ Ab responses	Many human vaccines (e.g., DTap, Hib, Hepatitis A, Hepatitis B)	Gavin et al. (2006), Franchi and Nùñez (2008), Kool et al. (2008a), McKee et al. (2009), Hutchison et al. (2012)
	NLRP3 activation *in vivo*?			
	Independent of TLR signaling			
	↑ Local cytokines and chemokines	↑ Th2 responses		
	↑ Cell recruitment (eosinophils, monocytes, macrophages)	Poor Th1 responses		
	↑ Ag presentation			
MF59	No depot effect	Balanced Th1 and Th2 responses	Licensed for influenza vaccine (Fluad^®^), H5N1 pre-pandemic vaccine (Aflunov^®^), H1N1 pandemic vaccines (Focetria^®^ and Celtura^®^)	Dupuis et al. (1999), Mosca et al. (2008), Calabro et al. (2011), Ellebedy et al. (2011)
	NLRP3 independent but ASC-dependent			
	Independent of TLR signaling but MyD88-dependent for Ab responses			
	↑ Local cytokines and chemokines			
	↑ Cell recruitment (neutrophils, macrophages, and monocytes)			
	↑ Ag uptake	
	Activate muscle cells			
	↑ Ag-loaded neutrophils and monocytes in dLNs			
AS04	MPL signals through TLR4 to activate APCs	↑ Ab responses	Licensed for human papilloma virus (HPV) (Cervarix™), hepatitis B virus (Fendrix^®^)	Didierlaurent et al. (2009)
	↑ Local cytokines and chemokines			
	↑ Cell recruitment (DCs and monocytes)	↑ Th1 responses		
	↑ Ag-loaded DCs and monocytes in dLNs			
AS03	Spatio-temporal co-localization with Ag	↑ Ab responses	Licensed for pandemic flu vaccine (Pandemrix^®^)	Morel et al. (2011)
	Transient ↑ cytokines locally and in dLNs			
	↑ Cell recruitment (granulocytes and monocytes)	↑ Immune memory		
	↑ Ag-loaded monocytes in dLNs			
Virosomes	Ag delivery vehicle	↑ Ab responses	Licensed for Inflexal^®^ V and Invivac^®^ influenza vaccine and hepatitis A vaccines (Epaxal^®^)	Glück et al. (1992), Bungener et al. (2002a,b), Khoshnejad et al. (2007)
	Bind APCs and induce receptor-mediated endocytosis	↑ CTL responses		
	Escape endosomal degradation			
	Ag presentation via MHC class II and MHC class I to CD4+ T cells and CD8+ T cells respectively			
	Immunopotentiator			

*Ab, antibody; Ag, antigen; CTL, cytotoxic T lymphocytes; dLNs, draining lymph nodes*.

## Formation of Depot at the Site of Injection

The formation of a depot at the injection site is perhaps the oldest and most widely recognized mechanism of action of adjuvants. Antigen trapping and slow release at the site of injection ensures constant stimulation of the immune system for production of high antibody titers (Siskind and Benacerraf, 1969). Until recently, depot effect was considered a classic mechanism of action of many adjuvants. Glenny et al. (1926) were the first to propose the importance of depot formation in the adjuvant activity of alum. Antigen was detected for 2–3 weeks in alumina gel-induced granulomas (Osebold, 1982). Antigens are simply adsorbed onto the alum but the binding is proposed to be due to strong electrostatic interaction between antigen and alum (Burrell et al., 2000), which enhanced antigen uptake and presentation by APCs (Mannhalter et al., 1985). Various other adjuvants such as water-in-oil emulsions [Complete Freunds Adjuvant (CFA)] and biodegradable micro-and nano-particles were shown to act by depot effect to generate prolonged and sustained high antibody titers (Herbert, 1968; Kreuter, 1988). AS04, an adjuvant combination consisting of monophosphoryl lipid A (MPL) and alum was shown to induce optimal immune responses only when co-localized with antigen (Didierlaurent et al., 2009). The presence of alum in AS04 is important in stabilizing the MPL and antigen within the vaccine, along with providing a depot effect (Didierlaurent et al., 2009). The cationic adjuvant formulation (CAF) 01, a combination of dimethyldioctadeclammonium/trehalose-6,6-dibehenate (DDA/TDB), which is currently in phase I clinical trial, is also thought to induce long lasting depot effect (Henriksen-Lacey et al., 2010).

There is no definitive evidence that depot effect significantly contributes to adjuvant activity (Marrack et al., 2009). In various studies, it has been shown that surgical removal of the antigen-alum depot 14 days after immunization had no effect on the immune responses (Schijns, 2000). Apparently, the adsorption of antigen to alum was not required for alum adjuvant activity (Iyer et al., 2003; De Gregorio et al., 2008). It was recently reported that removal of the injection site 2 h after antigen and alum administration had no effect on humoral or cell-mediated immunity (Hutchison et al., 2012). Similarly, MF59 was rapidly cleared and did not form a depot at the injection site (Ott et al., 1995). MF59 was distributed and cleared independent of antigen with a half-life of 42 h in the muscle tissue (Dupuis et al., 1999). Likewise, ISCOMs tend to be rapidly transported to the draining lymph nodes after administration (Morein and Bengtsson, 1999). Together, these studies clearly indicate that depot effect is not required for adjuvant activity of alum, and possibly MF59 or ISCOMs.

## Up-Regulation of Cytokines and Chemokines Leading to Cellular Recruitment at the Injection Site

Recent studies on the mechanisms of adjuvants have focused on recruitment of innate immune cells at the site of injection. Particulate adjuvants have been shown to create a local pro-inflammatory environment to recruit immune cells (Goto and Akama, 1982). Using genome wide microarray analysis, Mosca et al. (2008) demonstrated that a cluster of genes encoding cytokines, chemokines, innate immune receptors, interferon-induced genes, and gene encoding adhesion molecules defined as “adjuvant core response genes” were commonly modulated by alum, MF59, and CpG-ODN at the site of injection. Compared with alum and CpG-ODN (TLR9 agonist), MF59 was a strong modulator of adjuvant core response genes. Chemokines, which play a critical role in tissue specific migration of immune cells, were shown to be up-regulated by adjuvants at the injection site. MF59 significantly up-regulated the expression of CCR2, a receptor for CCL2, which is involved in monocyte infiltration. This was in agreement with previous *in vitro* results showing that MF59 induced release of chemo-attractants like CCL2, CCL3, CCL3 and CXCL8 (Seubert et al., 2008). Further, studies in CCR2-deficient mice showed that MF59-induced mononuclear cell recruitment is CCR2 dependent (Dupuis et al., 2001). Another oil-in-water emulsion AS03 co-localizes with antigen to trigger colony-stimulating factor 3 (CSF3) and IL-6, and leukocyte-recruiting chemokines CCL2, CCL3, and CCL5 at the site of injection (Morel et al., 2011). Similar cytokine and chemokine mRNA expression profiles were up-regulated in the draining lymph nodes (Morel et al., 2011). Likewise, alum-induced infiltration of immune cells was accompanied by production of chemo-attractants like CCL2, the neutrophil chemotaxin KC (CXCL1), and eosinophil chemotaxin eotaxin (CCL11) in the peritoneal cavity of mice (Kool et al., 2008b). Similarly, a novel adjuvant, poly[di(sodiumcarboxylatoethylphenoxy)phosphazene] (PCEP), induced stronger expression of adjuvant core response genes compared to CpG at the site of injection. Locally, PCEP triggered production of pro-inflammatory cytokines and chemokines including CCL2 (Awate et al., 2012).

Alum promotes Th2-type immune responses and differentiation of B cells resulting in robust antibody production (Grun and Maurer, 1989). However, the role of Th2 cytokines in the adjuvant activity of alum is not clearly defined. *In vitro* studies indicate that alum-induced activation of macrophages and up-regulation of co-stimulatory molecules did not depend on IL-4 (Rimaniol et al., 2004). However, in *in vivo* studies, alum-induced priming of B cells through IL-4 producing Gr1^+^ cells in mouse spleen, which is required for proliferation of antigen-specific B cells and for optimal antibody production (Jordan et al., 2004). IL-4 producing Gr1^+^ cells were mainly eosinophils, which appeared within 24 h and induced expansion of B cells and enhanced IgM production (Wang and Weller, 2008). Further, studies with eosinophil-deficient mice showed that the priming of B cells was abolished after alum injection confirming the central role of eosinophils in alum-induced Th2-type immune responses (Jordan et al., 2004; Wang and Weller, 2008). In addition, a study by Serre et al. (2008) revealed that the Th2-type immune responses generated by alum may signal through IL-25/IL-17RB and/or IL-6 pathways.

Alum has been shown to activate the complement cascade and recruit cells from blood to create an inflammatory environment at the site of injection (Ramanathan et al., 1979; Goto et al., 1997). Similar to alum, MF59 has been shown to recruit CD11b^+^ blood mononuclear cells in the mouse muscle (Mosca et al., 2008). Intra-peritoneal injection of alum-induced rapid cell recruitment of inflammatory Ly6C^+^CD11b^+^ monocytes. The inflammatory monocytes take up antigen, differentiate into CD11c^+^ MHC class II^+^ DCs in a myeloid differentiation primary response gene 88 (MyD88)-dependent manner and migrate to draining lymph nodes, where they induced proliferation of antigen-specific T cells (Kool et al., 2008a). In similar studies by McKee et al. (2009), alum-induced rapid recruitment of various polymorphonuclear (PMN) cells including eosinophils, monocytes, neutrophils, DCs, natural killer (NK), and NKT cells at the site of vaccination. Interestingly, in cell depletion studies in mice, alum-mediated humoral and cellular responses were independent of mast cells, macrophages, and of eosinophils (McKee et al., 2009).

MF59-mediated immune cell recruitment to the injection site has been studied in detail (Calabro et al., 2011). MF59 induced recruitment of neutrophils, monocytes, eosinophils, macrophages followed by DCs after i.m. injection in mice. The recruited cells especially neutrophils, monocytes, and B cells take up both antigen and adjuvant and traffick to draining lymph nodes. Neutrophils are the first cells to be recruited at the site of adjuvant injection and also one of the highest in numbers. However, depletion of neutrophils had no impact on the antigen-specific immune responses induced by MF59 (Calabro et al., 2011). Similar to MF59, administration of AS03 led to enhanced recruitment of neutrophils, eosinophils, and monocytes at the site of injection, which take up antigen and traffick to draining lymph nodes (Morel et al., 2011). At the injection site, neutrophils attract other immune cells by producing increased amounts of chemokines and transport antigen to the draining lymph nodes (Calabro et al., 2011; Morel et al., 2011). However, the role of neutrophils in adjuvant activity is not completely clear.

ASO4 induces transient local NFκB activity and cytokine production (Didierlaurent et al., 2009). The TLR4 agonist MPL, one of the components of AS04, stimulated increased numbers of DCs and monocytes in the draining lymph nodes. Likewise CpG, a TLR9 agonists, signals through activation of MyD88, IRAK, and TRAF-6, leading to recruitment of transcriptional factors, which in turn up-regulates the pro-inflammatory genes and protein expression (IL-1, IL-6, IL-12, IL-18, and TNF-α) within 3 h of injection (Klinman et al., 1996; Klaschik et al., 2009). Genes up-regulated by CpG included cytokines, cell signaling, cell movement, and DNA damage response genes (Klaschik et al., 2010). One of the roles of cationic liposomes is to recruit immune cells and increase antigen presentation. Intra-peritoneal injection of cationic liposome (DDA/MPL) increases influx of neutrophils, monocytes, macrophages, and activated NK cells in the peritoneal cavity (Korsholm et al., 2010). Another cationic liposome CAF01 induced recruitment of monocytes to the site of injection and increased trafficking of liposomes to the draining lymph nodes (Henriksen-Lacey et al., 2010).

Therefore, adjuvants induce recruitment of various immune cells to the site of injection, some of which then traffick the antigen to the draining lymph nodes to induce specific immune responses. However, the relationship between these recruited cells and induction of immune responses is not very clear. Depletion studies suggest that the role of recruited innate immune cells at the injection site is redundant in the generation of adaptive immune responses (McKee et al., 2009; Calabro et al., 2011). Interestingly, these studies were performed by depleting single cell populations. Identifying the role of a specific cell population *in vivo* is even more challenging due to complex environment at the injection site. Injection of adjuvants often leads to recruitment of a variety of cell populations and due to high redundancy in the immune system, other recruited cells may compensate for the depleted single cell population. In this regard, mice whose specific cell populations have been depleted were shown to produce cytokines and chemokines to recruit innate immune cells and activate T cells (Seubert et al., 2008; Calabro et al., 2011). Further studies are required to investigate the detailed relationship between recruited immune cells and adjuvant activity.

## Antigen Presentation

Efficient antigen presentation by MHCs on APCs is important for the induction of adaptive immune response. It was thought that many adjuvants including alum, oil-based emulsions, and microparticles act by “targeting” antigens to APCs resulting in enhanced antigen presentation by MHC (Guéry et al., 1996; Schijns and Lavelle, 2011). Alum was shown to increase antigen uptake by DCs and alter the magnitude and duration of antigen presentation (Mannhalter et al., 1985; Morefield et al., 2005). Antigen adsorption on alum led to an increase in internalization of antigen (Morefield et al., 2005). Recent studies by Flach et al. (2011) have shown that alum does not enter DCs directly but rather delivers the antigen via abortive phagocytosis. In this regard, alum interacts with membrane lipids on DCs leading to lipid sorting, recruitment of ITAM containing molecules Syk and PI3 activation. These events eventually lead to uptake of antigen that is adsorbed on alum, DC activation, and MHC class II expression (Flach et al., 2011).

The role of adjuvant-induced increased antigen presentation in development of adaptive immunity has not been clearly evaluated. Hence, our knowledge is limited regarding the role of this adjuvant mechanism. Recently, Ghimire et al. (2012) investigated the impact of antigen presentation on alum adjuvanticity. In addition to confirming the ability of alum to increase the antigen internalization, the study also showed that alum plays an important role in reducing the rate of degradation of internalized antigen (Ghimire et al., 2012). Similarly, MF59 facilitated internalization of gD2 antigen from type 2 herpes simplex virus (HSV) by recruited APCs at the site of injection and increased phagocytosis in human PBMCs (Dupuis et al., 1999). Antigen size seems to play an important role in modulating the antigen presentation efficiency. Large lipid vesicles end up in early endosome/phagosomes and increases antigen presentation whereas smaller vesicles rapidly localize to late lysosomes leading to reduced antigen presentation (Brewer et al., 2004).

## Activation and Maturation of DCs

Activation of DCs is essential for induction of adaptive immune responses. Increased expression of MHC class II, activation marker CD86, and maturation marker CD83 leads to enhanced ability of APCs to induce T lymphocyte activation and differentiation (Coyle and Gutierrez-Ramos, 2001). Freund’s complete adjuvant, lipopolysaccharide (LPS), liposomes, CpG-ODN, MF59, AS04, and α-galactosylceramide (α-GAL) have all been shown to induce DC maturation to enhance adaptive immunity (De Smedt et al., 1996; De Becker et al., 2000; Copland et al., 2003; Fujii et al., 2003; Shah et al., 2003). Intra-peritoneal injection of OVA and alum led to uptake of antigen and maturation of DCs (Kool et al., 2008a). However, *in vitro* studies on human cells have shown that alum and MF59 failed to directly activate DCs but enhanced the surface expression of MHC class II and co-stimulatory molecules (CD83 and CD86) on monocytes, macrophages, and granulocytes that resulted in increased T cell proliferation (Sun et al., 2003; Seubert et al., 2008). Further, *in vitro* activation of DCs by alum has generated conflicting results. One study suggested that alum failed to induce maturation and antigen presentation (Sun et al., 2003) where as another study showed that the activation marker CD86 and antigen presentation was increased in DCs (Sokolovska et al., 2007). The source of alum may have been a contributing factor in the conflicting results.

AS04 has been shown to induce maturation of DCs (via TLR4), which then trafficks to the draining lymph nodes to activate antigen-specific T cells (Didierlaurent et al., 2009). Similarly, CpG induced up-regulation of CD40, CD54, CD80, CD86, and MHC class II molecules and antigen processing and presentation in plasmacytoid DCs (pDCs) (Krieg, 2002; Kerkmann et al., 2003). A novel class of TLR-independent adjuvants, mycobacterial cord factor trehalose-6-6-dimycolate (TDM) and TDB have been shown to directly activate DCs through the FcγR-Syk-Card9-Bcl10-Malt1 pathways, and up-regulates the expression of co-stimulatory molecules (Werninghaus et al., 2009). Microparticles such as Poly-lactic-co-glycolic acid (PLGA) did not induce co-stimulatory molecules expression on bone marrow derived DCs (BMDCs) but enhanced antigen presentation efficiency (Sun et al., 2003). DOTAP (1,2-dioleoyl-3-trimethylammonium-propane)-based cationic liposomes have been shown to induce maturation of DCs through activation of MAPK (extracellular signal-regulated kinase and p38), leading to up-regulation of co-stimulatory molecules (Yan et al., 2007). Likewise, diC14-amidine (3-tetradecylamino-tert-butyl-N-tetradecylpropion-amidine) based cationic liposomes up-regulates the expression of CD80 and CD86 on DCs through specific TLR4/MD2 ligation (Tanaka et al., 2008). Overall, adjuvants stimulate DC maturation and enhance the expression of MHC and co-stimulatory molecules, which is required for efficient T cell activation.

## Activation of Inflammasomes

Innate immune cells express various pathogen-recognition receptors (PRRs) to recognize infectious agents. In recent years, various new families of PRRs have been identified including TLRs, C-type lectin-like receptors (CLRs), nucleotide oligomerization domain (NOD) like receptors (NLRs), and Retinoic acid-inducible gene-1 (RIG-1) like receptors (RLRs). Many immunological adjuvants signal via PRRs or act as ligands for innate immune receptors (Table 2). In contrast to TLR agonists, particulate adjuvants are not recognized by specific PRRs but they still induce adaptive immune responses. The “danger” hypothesis was first advanced by Matzinger (1994), who proposed that apart from self/non-self discrimination against infection, danger signals from damaged cells can trigger activation of the immune system. Molecules associated with tissue damage such as uric acid, nucleotides, adenosine triphosphate (ATP), reactive oxygen intermediates, and cytokines are released at the injection site due to tissue damage (Shi et al., 2003). These non-infectious damage signals have now been named damage-associated molecular patterns (DAMPs) to distinguish them from pathogen-associated molecular patterns (PAMPs).

**Table 2:** **Innate immune receptors activated by vaccine adjuvants**.

PRRs		Adjuvants	Type of immune response induced	Reference
TLRs	TLR1/2	Triacyl lipopeptides	Th1, Th2, CTL responses	Deres et al. (1989), Schild et al. (1991)
		Synthetic Pam_3_Cys	
	TLR2/6	Diacyl lipopeptides	Th1, Th2, CTL responses	Moyle and Toth (2008)
		Pam_2_Cys	
	TLR2	Pam_3_Cys	Th1, Th2, CTL responses	Deres et al. (1989), Schild et al. (1991)
	TLR3	Poly I:C	Both Th1 and Th2	Tamura and Sasakawa (1983), Choi et al. (2012)
	TLR4	LPS, AS04 (MPL)	Th1	Sasaki et al. (1997), Casella and Mitchell (2008)
	TLR5	Flagellin	Th1 and Th2	Didierlaurent et al. (2004), McCarron and Reen (2009)
	TLR 7	Imiquimod	Th1, CD8^+^ T cell, CTL responses	Wagner et al. (1999), Stanley (2002)
		Resiquimod	
	TLR8	Resiquimod	Th1, CD8^+^ T cell, CTL responses	Wagner et al. (1999), Wu et al. (2004)
	TLR9	CpG-ODN	Th1, CD8^+^ T cells, CTL responses	Kobayashi et al. (1999)
NLRs	NOD1/NLRC1	DAP	Th1, Th2, Th17	Chamaillard et al. (2003), Girardin et al. (2003a), Fritz et al. (2007)
	NOD2/NLRC2	MDP	Th1, Th17	Girardin et al. (2003b), van Beelen et al. (2007), Shaw et al. (2009)
	NLRP1	Toxoids, MDP	Th1	Hsu et al. (2008)
	NLRP3	Alum, MDP, ATP	Th2	Mariathasan et al. (2006), Li et al. (2007), Eisenbarth et al. (2008)
	IPAF/NLRC4	Flagellin	Th1 and Th2	Lightfield et al. (2011), Zhao et al. (2011)
	NAIP5	Flagellin	Th1 and Th2	Kofoed and Vance (2011)
RLRs	RIG-1	DNA vectors	Th1, CD8^+^ T cells	Luke et al. (2011)
	MDA5	Poly I:C	Th1, CD8^+^ T cells	Wang et al. (2010)
CLRs	Dectin-1	Flagellin, β-glucan/zymosan	Th17	LeibundGut-Landmann et al. (2007)
	Mincle	CAF01	Th1, Th17 CD8^+^ T cells	Gram et al. (2009), Rosenkrands et al. (2011)

*Pam3Cys, tri-palmitoyl-S-glyceryl cysteine; LPS, lipopolysaccharide; AS04, adjuvant system 04; MPL, monophosphoryl lipid A; CpG-ODN, cytidine-phosphate-guanosine oligodeoxynucleotides; Poly I:C, polyinosinic-polycytidylic acid; DAP, diaminopimelic acid; MDP, muramyl dipeptide; CAF01, cationic adjuvant formulation-01; TLR, toll-like receptor; NLR, NOD-like receptors; RLR, RIG-1 like receptors; CLR, C-type lectins; NOD, nucleotide-binding oligomerization domain; NLRP3, NOD-like receptor family, pyrin-domain-containing 3; IPAF, IL-1β-converting enzyme protease-activating factor; NAIP, neuronal apoptosis inhibitory protein; RIG-1, retinoic acid-inducible gene-1; MDA5, melanoma differentiation associated gene 5*.

Particulate adjuvants cause local tissue damage and cell death at the injection site (Kool et al., 2008a). In addition, many adjuvants induce release of pro-inflammatory cytokines at the site of injection (Didierlaurent et al., 2009; Calabro et al., 2011; Awate et al., 2012). These damage signals trigger non-specific activation of the innate immune system, subsequently stimulating adaptive immunity. Recently inflammasomes have been one of the most widely investigated topics due to their potential role in adjuvant activity. The inflammasome belongs to the NLR family, which also includes various other receptors, such as the NODs (NOD1-5), NLRPs (NLRP1-14), NLRP1 (NAIP), NLRC4 (IPAF), and the major histocompatibility complex II transactivator (CIITA) (Martinon et al., 2009). Compared to others, NOD-like receptor family, pyrin-domain-containing 3 (NLRP3) is the most studied inflammasome receptor in regards to adjuvant mechanisms. NLRP3, also known as cryopyrin or NALP3 (NACHT, LRR, and PYD domains-containing protein 3), is an intra-cytoplasmic multi-protein complex that consists of three components; a NLRP3 receptor, an apoptosis-associated speck-like protein containing a CARD (ASC) and a procaspase-1 (Schroder and Tschopp, 2010). Activation of NLRP3 inflammasome induces caspase-1 activation, which in turn cleaves proforms of IL-1β, IL-18, and IL-33 to their bioactive forms (Martinon et al., 2009). The NLRP3 inflammasome can be activated by various stimuli including DAMPs, environmental irritants such as asbestos and silica, metabolic stress, and UVB irradiation (Schroder and Tschopp, 2010). Apart from danger signals, inflammasomes can be activated by PAMPs such as bacterial flagellin through NLRC4 activation (Zhao et al., 2011).

Li et al. (2007) reported for the first time that alum-induced secretion of IL-1β and IL-18 was caspase-1 dependent. Subsequent *in vitro* studies by various groups showed that activation of NLRP3 is required for alum-induced IL-1β and IL-18 secretion (Eisenbarth et al., 2008; Franchi and Nùñez, 2008; Hornung et al., 2008; Kool et al., 2008a). However, LPS priming to induce pro-IL-1β in APCs prior to alum stimulation was a pre-requisite for secretion of IL-1β. Contrary to *in vitro* studies, the role of inflammasomes in the adjuvant activity of alum *in vivo* has yielded conflicting results. Using NLRP3, ASC and caspase-1 knockout mice, Eisenbarth et al. (2008) showed that the NLRP3 inflammasome is a crucial component in the adjuvant activity of alum. NLRP3, ASC, and caspase-1 knockout mice immunized with OVA adsorbed on alum, failed to induce antigen-specific antibody responses (Eisenbarth et al., 2008). Another study by Kool et al. (2008a) showed that alum-induced lower influx of inflammatory cells in the peritoneal cavity of NLRP3 deficient mice. They also showed that alum-mediated activation of adaptive immune responses was NLRP3-dependent (Kool et al., 2008a). Similar studies done by Li et al. (2008) showed that NLRP3 deficient mice injected with alum-adsorbed diphtheria toxoid or OVA vaccine elicited impaired levels of antigen-specific antibody responses. All these studies indicate that NLRP3 inflammasome is critical in the adjuvant activity of alum *in vivo*. In contrast, Franchi and Nùñez (2008) clearly showed that antigen-specific IgG production was not impaired in NLRP3 deficient mice following intra-peritoneal injection of human serum albumin (HSA) in the presence of alum. However, NLRP3 did affect alum-mediated cellular recruitment suggesting that inflammasomes might play an important role in activating innate immunity, but the contribution of inflammasomes in activation of adaptive immunity remains elusive. The conflicting results with regard to the role of inflammasomes in adjuvant activity of alum have been attributed to the differences in the nature of alum used in different studies, immunization protocols, and the mouse strains used (De Gregorio et al., 2008; Marrack et al., 2009).

To date, the ligand for NLRP3 has not been identified. Some theories proposed for alum-mediated activation of NLRP3 include phagosomal destabilization and release of cathepsin B, low intracellular potassium (K+) concentrations, and generation of reactive oxygen species (ROS) (Petrilli et al., 2007; Hornung et al., 2008; Kool et al., 2008a). It was proposed that a catabolic product of nucleotides, uric acid, and ATP released at the site of alum injection due to cell damage or necrosis act as danger signals for activation of NLRP3. Saturation of uric acid due to tissue damage forms mono-sodium ureate crystals (MSU). Phagocytosis of crystalline particles such as MSU or alum results in phagosomal destabilization and lysosomal rupture releasing the protease cathepsin B in the cytosol (Hornung et al., 2008). The released cathepsin B led to activation of NLRP3 and secretion of pro-inflammatory cytokines IL-1β and IL-18. Treatment of mice using uricase, a uric acid degrading enzyme, led to reduced cellular recruitment to draining lymph nodes in mice injected with alum (Kool et al., 2008a). Similarly, ATP released by the damaged cells at the injection site has been shown to indirectly activate NLRP3. Extracellular ATP triggered stimulation of purinergic P2X_7_ receptor, resulting in activation of cation channel for K+ efflux and opening of pannexin-1 pore for entry of danger signals generated by alum, activate NLRP3 and subsequently caspase-1 (Solle et al., 2001; Petrilli et al., 2007). Further, blocking ROS using chemical scavengers abolished NLRP3 activation in response to MSU suggesting a link between NLRP3 activation and ROS generation (Dostert et al., 2008).

Recently, the role of the inflammasome in adjuvant activity of MF59 was evaluated (Ellebedy et al., 2011; Seubert et al., 2011). Two independent studies using NLRP3 deficient mice demonstrated that NLRP3 is not required for the adjuvant activity of MF59. However, an adaptor molecule required for the assembly of inflammasome, ASC was found to be crucial for MF59 adjuvant activity (Ellebedy et al., 2011). A recent study by Embry et al. (2011) showed that MPL failed to induce intra-cytoplasmic assembly of NLRP3 inflammasome leading to failure of caspase-1 activation and maturation of pro-inflammatory cytokines IL-1β and IL-18.

## Conclusion

The ultimate goal of vaccination is to generate protection against disease causing pathogens. Protective immunity against different pathogens requires different immune responses that can be generated by using appropriate vaccine adjuvants. Therefore, a detailed knowledge of the mechanisms of action of adjuvants is very important in the rational design of vaccines. In recent years, considerable advances have been made in understanding the mechanisms of action of various adjuvants, particularly the activation of innate immunity via various mechanisms (Table 1). The future of vaccine adjuvant research is heading toward developing novel combination adjuvants that consist primarily of PRRs agonists and particulate adjuvants. While combining different adjuvants results in potent formulation that can enhance the quality and quantity of immune response against vaccine antigens, adjuvant combinations may also have more complex mechanisms of action.

Safety is a major concern when it comes to adjuvant approval for human use. Detailed understanding of the mechanisms of action of adjuvants will provide some insight into their safety. In addition, since all of the adjuvants approved and currently in clinical trials are in vaccines administered by injection, there is a need to identify and develop good mucosal adjuvants. In the coming years, we hope to learn more details of the various mechanisms of action of adjuvants, which will be valuable in rational vaccine design and hopefully lead to approval of new adjuvants for use in vaccines for humans.

## Conflict of Interest Statement

The authors declare that the research was conducted in the absence of any commercial or financial relationships that could be construed as a potential conflict of interest.
